# Compound 49b protects against blast-induced retinal injury

**DOI:** 10.1186/1742-2094-10-96

**Published:** 2013-07-30

**Authors:** Youde Jiang, Li Liu, Jayaprakash Pagadala, Duane D Miller, Jena J Steinle

**Affiliations:** 1 Department of Ophthalmology, Hamilton Eye Institute, 930 Madison Ave, Suite 768A, Memphis, TN 38163, USA; 2 Department of Pharmaceutical Sciences, University of Tennessee Health Science Center, Memphis, TN 38163, USA; 3 Department of Anatomy & Neurobiology, University of Tennessee Health Science Center, Memphis, TN 38163, USA

**Keywords:** Apoptosis, Beta-adrenergic receptor agonists, Cytokines

## Abstract

**Aim:**

To determine whether Compound 49b, a novel beta-adrenergic receptor agonist, can prevent increased inflammation and apoptosis in mice after exposure to ocular blast.

**Methods:**

Eyes of C57/BL6 mice were exposed to a blast of air from a paintball gun at 26 psi (≈0.18 MPa). Eyes were collected 4 hours, 24 hours, and 72 hours after blast exposure. In a subset of mice, Compound 49b eyedrops (1 mM) were applied within 4 hours, 24 hours, or 72 hours of the blast. Three days after blast exposure, all mice were sacrificed. One eye was used to measure levels of retinal proteins (TNFα, IL-1β, Bax, BcL-xL, caspase 3, and cytochrome C). The other eye was used for TUNEL labeling of apoptotic cells, which were co-labeled with NeuN to stain for retinal ganglion cells.

**Results:**

We found that ocular exposure to 26 psi air pressure led to a significant increase in levels of apoptotic and inflammatory mediators within 4 hours, which lasted throughout the period investigated. When Compound 49b was applied within 4 hours or 24 hours of blast injury, levels of apoptotic and inflammatory mediators were significantly reduced. Application of Compound 49b within 72 hours of blast injury reduced levels of inflammatory mediators, but not to untreated levels.

**Conclusions:**

Ocular blast injury produces a significant increase in levels of key inflammatory and apoptotic markers in the retina as early as 4 hours after blast exposure. These levels are significantly reduced if a beta-adrenergic receptor agonist is applied within 24 hours of blast exposure. Data suggest that local application of beta-adrenergic receptor agonists may be beneficial to reduce inflammation and apoptosis.

## Background

Ocular trauma constitutes one of the most common causes of unilateral morbidity and blindness in the world today [1]. During recent wars, many ocular injuries have been caused by explosions with fragmentary munitions; they are the fourth most common injury in U.S. Operation Iraqi Freedom [2]. Owing to improvements in body protective gear, the rates of combat-based morbidity and mortality have decreased, while the number of ocular injuries has increased (from 0.57% during the American Civil War to 13% in U.S. Operation Desert Storm) [1,3]. While all soldiers agree that eyewear is important, many are noncompliant because the eyewear becomes foggy, is bulky, or is unstylish [2]. In addition to the compliance issue, the ability of eye protective gear is, at present, limited. Even with improved eye protective wear, eye injuries still occur in 24% of blast injury cases. Thus, despite developments in military protective wear, the blast produced by many improvised explosive devices is associated with closed, as well as open-globe, injuries from fragmentary munitions. Owing to other life-threatening injuries that may occur after exposure to blasts from such devices, ocular repair and treatment is often delayed for as long as 3 or 4 days after the initial injury [2,3].

To better understand the nature of eye damage after exposure to ocular blast, a good model needs to be developed. Unfortunately, before this year, few such models have existed. Whole-body models of blast injury have been used to investigate the effects of blasts on major organ systems [4] or to the brain [5]. These studies demonstrated that Kevlar protection is effective in protecting internal organs from injury, but that the brain and eyes are still affected by the blast wave. Furthermore, although work in the brain-blast model [6] demonstrated damage to the visual tracts of the brain, the retina itself was not fully examined. To better mimic ocular trauma and allow for thorough characterization of retinal responses, a new model has been developed using an air blast from a paintball gun as the primary inducer of trauma [7]. Using this model, Hines-Beard *et al*. demonstrated with high-resolution optical coherence topography, gross pathology, and optokinetics that a pressure of 23 to 26 psi (≈0.16 to 0.18 ≈ MPa) produced a number of anterior and a few posterior ocular injuries [7]. In this study of various blast pressures, the authors found only one eye to have changes to the retina, choroid, or retinal pigmented epithelium after exposure to 26 psi. This corresponds well with previous studies in veterans [8]. Despite the lack of gross pathology, it is probable that the posterior eye is still altered after blast exposure, and will produce increased levels of inflammatory or apoptotic markers. To investigate changes in retinal inflammatory and apoptotic mediators after blast, we employed the same model as described in [7] and measured levels of key proteins within 4 hours, 1 day, and 3 days after blast exposure.

Furthermore, we have previously reported that β-adrenergic receptor agonists, particularly a novel drug, Compound 49b, have anti-apoptotic and anti-inflammatory properties in retinal endothelial cells and in a diabetic retinopathy model [9,10]. Compound 49b is based structurally on isoproterenol, with chemical modifications to increase its ocular potency as a topical treatment. The chemical properties of Compound 49b are listed in Table 1. The chemical structure of Compound 49b is patent pending [11]. Our hypothesis in this study was that topical application of Compound 49b within 24 hours of blast injury would prevent blast-induced increases in inflammatory mediators and apoptotic markers.

**Table 1:** Compound 49b

Molecular formula (in salt)	C_19_H_26_ClNO_6_
Molecular weight (in salt)	399.87
Molecular formula (in neutral)	C_19_H_25_NO_6_
Molecular weight (in neutral)	363.40
Octanol-water partition coefficient, log *P*	1.92
pKa	≈ 9

Octanol-water partition coefficient (log *P*) of Compound 49b is a measure of its lipophilicity. Log *P* was calculated using ChemDraw Ultra version 8.0 (CambridgeSoft Corporation, Cambridge, MA).

## Methods

### Mice

C57/BL6 mice were purchased from Charles River (Wilmington, MA) at 2 months of age. Mice were exposed to an ocular blast of 26 psi (≈0.18 MPa) to both eyes using an air blast from a paintball gun [7]. The pressures were measured and analyzed using Labview software (National Instruments, Austin, TX). All animal experiments were approved by the Institutional Animal Care and Use Committee at the University of Tennessee Health Science Center (Protocol #2079).

Both eyes of the mice were exposed to the ocular blast. In one subset of mice, eyes were collected at 4 hours post-blast, 24 hours post-blast, 72-hours post-blast, or at 72 hours without exposure to the blast. In a second subset of mice, both eyes were blasted; however, a novel β-adrenergic receptor agonist, Compound 49b (1 mM), was applied topically within 4 hours, 24 hours, or 72 hours post-blast. The mice treated with Compound 49b received Compound 49b treatment daily for up to 3 days. For example, for the 4-hour treatment group, the mice received the first treatment within 4 hours post-blast, then further treatments at 24 hours, 48 hours, and 72 hours post-blast (for a total of four treatments), while the mice who were treated 72-hours post-blast only received one treatment of Compound 49b prior to sacrifice. All mice treated with Compound 49b were sacrificed three days post-blast. Ten mice were used at each time point for all experiments.

### Western blotting

At the appropriate time after the blast or Compound 49b treatments, one eye was used for protein analyses. For Western blot analyses, retinal lysates were collected into lysis buffer containing protease and phosphatase inhibitors and scraped into the tubes. Retinal extracts were prepared by sonication. Equal amounts of protein from the cell or tissue extracts were separated on pre-cast tris-glycine gel (Invitrogen, Carlsbad, CA), blotted onto a nitrocellulose membrane. After blocking in Tris-buffered saline with Tween 20 (10 mM Tris–HCl buffer, pH 8.0, 150 mM NaCl, 0.1% Tween 20) and 5% (w/v) BSA, the membrane was treated with appropriate primary antibodies followed by incubation with secondary antibodies labeled with horseradish peroxidase. Antigen-antibody complexes were detected using a chemiluminescence reagent kit (Thermo Scientific). Primary antibodies used were cytochrome C, Bax, and BcL-xL, NFkB, and phosphorylated NFkB (all purchased from Cell Signaling, Danvers, MA).

### ELISA

A cleaved caspase 3 ELISA (Cell Signaling, Danvers, MA) was used to measure levels of the active apoptotic markers in whole retinal lysates. TNFα and IL-1β protein concentrations were measured using TNFα and IL-1β ELISAs, respectively (ThermoFisher, Pittsburgh, PA). All ELISAs were done according to the manufacturer’s instructions with equal protein loaded into all wells.

### Terminal deoxynucleotidyl transferase mediated dUTP nick end labeling assay (TUNEL)

TUNEL was done on 10 μm cryosections of mouse retina according to the manufacturer’s instructions using the ApopTag-FITC kit (Millipore, Bilerica, MA) to localize apoptotic cells in the retina at the various time points. TUNEL positive cells were co-localized with NeuN antibody (Abcam), to demonstrate which cell types were undergoing apoptosis.

### Statistics

For all analyses, all experiments were done in triplicate. Data are presented as mean ± standard error of the mean, with statistical analyses using Kruskal-Wallis nonparametric testing, followed by Dunn’s test.

## Results

### Blast exposure increases TNFα and IL-1β levels in retinal lysates as early as 4 hours; this is mitigated by topical Compound 49b

**Figure 1:**
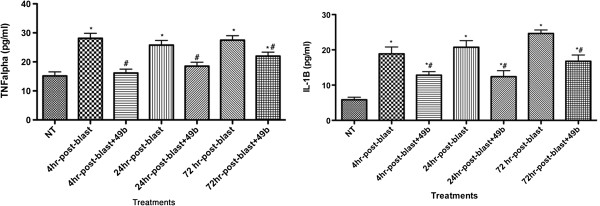
**ELISA results for TNFα (left) and IL-1β (right) in mouse retina without exposure to blast (NT) or with exposure to blast for 4, 24, or 72 hours with or without Compound 49b for 4, 24, or 72 hours.** *P < 0.05 vs. NT. #*P* < 0.05 vs. blast only at the same time point. *N* = 5 mice for each group. NT, no treatment.

Since a thorough analysis of inflammatory and apoptotic markers after blast exposure has not been reported, we chose to investigate levels of TNFα and IL-1β, both key proteins in other retinal diseases [12,13], within 4 hours, 24 hours, and 72 hours of exposure to 26 psi (≈0.18 MPa) blast. We found that protein levels of both TNFα and IL-1β were significantly increased within 4 hours of blast exposure and remained elevated for the 72 hours investigated (Figure 1). Additionally, we found that if topical Compound 49b (1 mM) was applied within 4, 24, or 72 hours of the blast exposure, the β-adrenergic receptor agonist could significantly reduce levels of both TNFα and IL-1β (Figure 1). In the case of TNFα, treatments within 4 or 24 hours of blast exposure could return TNFα levels to a level not significantly different from that for an eye that had not been exposed to a blast.

### Compound 49b reduces apoptotic proteins after exposure to ocular blast

**Figure 2:**
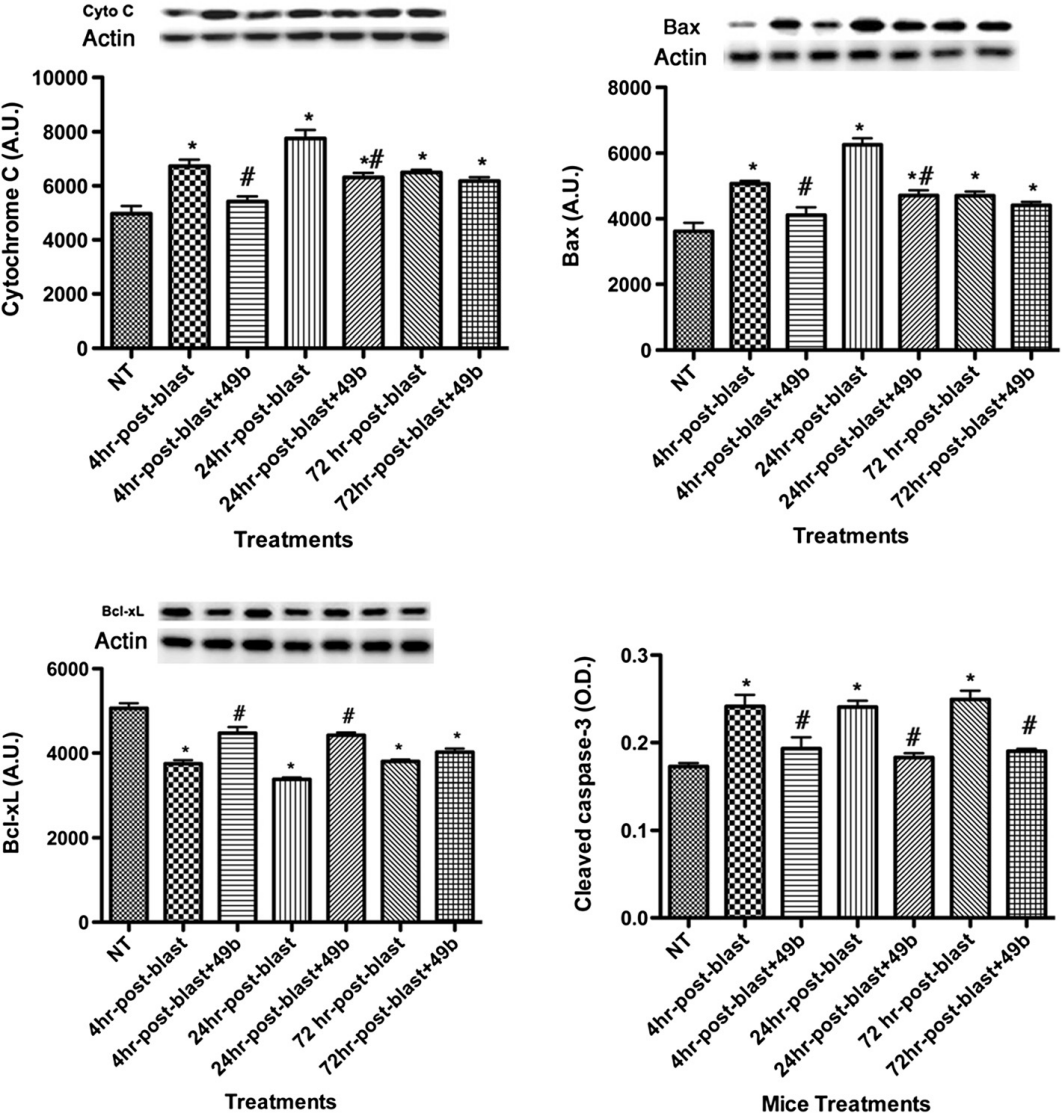
**Western blot results for key pro-apoptotic proteins (Cytochrome C and Bax—top) and anti-apoptotic protein BcL-xL (bottom left).** ELISA results for cleaved caspase 3. *P < 0.05 vs. NT. #*P* < 0.05 vs. blast only at the same time point. *N* = 5 mice for each group. NT, no treatment.

Because activation of inflammatory mediators often leads to apoptosis [14,15], we evaluated levels of key apoptotic proteins (Bax, cytochrome C, cleaved caspase 3) and an anti-apoptotic marker (BcL-xL) after exposure to blast alone or blast + Compound 49b at 4, 24, and 72 hours post-blast (Figure 2). Blast exposure significantly increased levels of pro-apoptotic markers and reduced levels of BcL-xL. Compound 49b effectively reduced Bax and cytochrome C levels and increased BcL-xL levels if applied within 24 hours of blast. Compound 49b was effective in reducing cleaved caspase 3 at all times investigated. Taken together, this demonstrates that the blast induces a strong apoptotic response, which is mitigated by application of Compound 49b, best applied within 1 day of blast exposure.

### Ocular blast induces apoptosis of cells in ganglion cell layer

**Figure 3:**
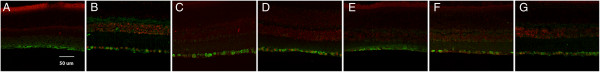
**TUNEL labeling and NeuN (retinal ganglion cell marker). (A)** untreated mice, **(B)** 4 hours post-blast, **(C)** 4 hours post-blast with Compound 49b, **(D)** 24 hours post-blast, **(E)** 24 hours post-blast with Compound 49b, **(F)** 72 hours post-blast, and **(G)** 72 hours post-blast with Compound 49b. TUNEL labeling is green; NeuN staining is red. Scale bar is 50 μm.

To visualize cells undergoing apoptosis after ocular blast exposure, we performed TUNEL labeling of retina sections with co-localization with NeuN to label retinal ganglion cells after blast with or without Compound 49b (Figure 3). It is clear that apoptosis is occurring in cells of the ganglion cell layer as early as 4 hours after blast, and becoming more pronounced over the 72 hours of the study. Compound 49b reduces this apoptosis; this finding is in agreement with the apoptotic protein marker analyses.

## Discussion

Ocular trauma is a leading cause of vision loss for soldiers, as well as the general public [3,16]. Unfortunately, little is known of the effects of exposure of blast pressure to the retina. Using the same model as used for this work, Hines-Beard only observed changes to the retinal pigmented epithelium in one eye [7]. While the morphology of the retina might not have changed, protein expression within the retina would probably have become activated and initiated changes, to be manifested in the morphology in the future. We found that within 4 hours of exposure to an ocular blast, a significant increase in levels of key inflammatory and apoptotic markers could be observed. This was associated with increased TUNEL labeling within the cells of the ganglion cell layer, which became more pronounced with additional exposure time of inflammatory and apoptotic markers.

Little is known on cellular changes following exposure to ocular blast or in closed-globe ocular injuries. For most ocular trauma studies, work has focused on corneal burns or trauma. However, since it is likely that other ocular targets are affected following exposure to the blast, literature on cellular changes in these targets may be relevant. Mice receiving a chemical burn to the cornea had significantly increased levels of TNFα and IL-1β, as well as macrophage migration inhibitory factor [17]. In a review of animal models of retinal injury, retinal ganglion cell apoptosis and inflammation are key points of discussion [18]. While the majority of the discussion in the work by Blanch *et al.*[18] was focused on axotomy of retinal ganglion cells or the optic nerve, the findings are similar to our observations after exposure to ocular blast, with increased levels of inflammatory mediators and apoptotic rates. In a subsequent paper on retinal changes in a closed-globe injury model, these authors describe increased TUNEL labeling and apoptosis of photoreceptors in the retina, after injury had been induced by firing an air gun pellet or ball bearing into mice’s eyes [19]. In this model involving projectiles into the eye, photoreceptor apoptosis and necrosis were observed, but specific apoptotic proteins or inflammatory proteins were not investigated. It is clear that further work on the cellular changes in the retina after injury is warranted.

Our findings of increased inflammatory and apoptotic markers after exposure to ocular blast agree with work from corneal burns or other closed-globe models. A recent report on British soldiers in Iraq and Afghanistan investigated ocular injuries, with the primary injury being trauma from exposure to a foreign body. In that work, the authors concluded that treatments could be safely delayed for 24 hours to allow for treatment of more life-threatening injuries [20]. Our results with Compound 49b eyedrop therapy support this conclusion, demonstrating that Compound 49b can reduce both inflammatory mediators and apoptotic markers for up to 72 hours after blast exposure. We have previously reported that Compound 49b is effective in reducing levels of TNFα and apoptotic proteins in diabetic animals up to 6 months, when applied daily [9]. Isoproterenol was equally effective at reducing levels of TNFα and apoptosis, but it had unwanted cardiovascular effects [10]. Future work will focus on the mechanisms by which Compound 49b can reduce inflammatory and apoptotic markers induced by ocular blast exposure.

## Conclusions

Exposure to ocular blast, similar to closed-globe injuries observed in soldiers, increases key inflammatory and apoptotic proteins for up to 72 hours after blast exposure. This response occurred primarily in the cells of the ganglion cell layer of the retina. Compound 49b, a novel β-adrenergic receptor agonist, could mitigate the increased inflammatory and apoptotic markers, with optimal responses observed when treatment was initiated within at least 24 hours of blast exposure. Since Compound 49b has little observed toxicity and can be administered in an eyedrop, it may offer a new therapy to protect the retina of soldiers after exposure to explosive devices in the combat field.

## Abbreviations

BSA: Bovine serum albumin; ELISA: Enzyme-linked immunosorbent assay; IL-1β: Interleukin-1β; TNFα: Tumor necrosis factor alpha; TUNEL: Terminal deoxynucleotidyl transferase mediated dUTP nick end labeling assay.

## Competing interests

YJ, DDM, and JJS are inventors of Compound 49b for blast injury.

## Authors’ contributions

YJ completed all experiments and analyzed the data. LL completed the immunohistochemical staining. DDM, JP, and JJS designed Compound 49b. JJS designed the experiments, assisted with data analysis, and wrote the paper. All authors have read and approved the final manuscript.
